# Surface engineering in PbS *via* partial oxidation: towards an advanced electrocatalyst for reduction of levulinic acid to γ-valerolactone†
†Electronic supplementary information (ESI) available. See DOI: 10.1039/c8sc03161d


**DOI:** 10.1039/c8sc03161d

**Published:** 2018-12-03

**Authors:** Haoran Wu, Jinliang Song, Chao Xie, Yue Hu, Pei Zhang, Guanying Yang, Buxing Han

**Affiliations:** a Beijing National Laboratory for Molecular Science , CAS Key Laboratory of Colloid and Interface and Thermodynamics , CAS Research/Education Center for Excellence in Molecular Sciences , Institute of Chemistry, Chinese Academy of Sciences , Beijing 100190 , China . Email: songjl@iccas.ac.cn ; Email: hanbx@iccas.ac.cn; b School of Chemistry and Chemical Engineering , University of Chinese Academy of Sciences , Beijing 100049 , China

## Abstract

Development of mild and efficient strategies for biomass conversion is of great significance, and design of advanced catalysts is crucial for biomass valorization. Herein, we designed PbS-based electrocatalysts through a surface engineering strategy *via* partial oxidation, and the degree of surface oxidation of PbS to PbSO_4_ could be easily tuned by calcination temperature. It was discovered that the prepared electrocatalysts could efficiently catalyze reduction of biomass-derived levulinic acid (LA) to γ-valerolactone (GVL) using water as the hydrogen source. Especially, the electrocatalyst calcined at 400 °C (PbS-400) showed outstanding performance with a current density of 13.5 mA cm^–2^ and a GVL faradaic efficiency of 78.6%, which was far higher than the best results reported up to date. Moreover, GVL was the only product from LA reduction, indicating the excellent selectivity. Mechanism investigation showed that LA was converted through electrocatalytic hydrogenation of carbonyl groups of LA and subsequent intramolecular esterification.

## Abstract 2

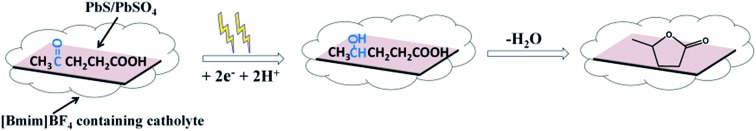
PbS-based materials *via* partial oxidation showed excellent performance for electroreduction of biomass-derived levulinic acid with a high GVL faradaic efficiency of 78.6% at a current density of 13.5 mA cm^–2^.

## Introduction

Transformation of renewable biomass into value-added chemicals is of great importance for a sustainable future, and has received tremendous interest.^1^–^5^ In this respect, hydrogenation of levulinic acid (LA) generated from lignocellulosic biomass into γ-valerolactone (GVL) is one of the most attractive reactions because GVL is a versatile chemical with diverse applications.^6^–^9^ Compared with thermal hydrogenations,^10^–^13^ electrochemical reduction of LA into GVL under mild conditions using H_2_O as the hydrogen source is a promising strategy. For example, Xin and co-workers used a lead electrode for electrochemical reduction of LA in KH_2_PO_4_/K_2_HPO_4_ electrolyte, obtaining a low GVL faradaic efficiency (6.2%) but with a high selectivity (100%).^14^ Uwe *et al.* carried out the electrocatalytic reduction of LA to GVL over iron electrodes in 1 M NaOH electrolyte to achieve a GVL faradaic efficiency of 20% with a selectivity of 70%.^15^ However, to obtain high faradaic efficiency and selectivity simultaneously for the electrochemical reduction of LA to GVL is highly challenging.

Metal chalcogenides (*e.g.*, MoS_2_, Cu_2_S, PbS) have attracted much attention owing to their outstanding mechanical, electrical, and optical properties,^16^–^19^ and these properties can be easily tuned by engineering the surface and interface for diverse applications.^20^–^23^ As an attractive approach of interface engineering, changing the surface composition through chemical or physical methods provides many possibilities for modifying the properties of metal chalcogenides by interfacial effects. For electrochemical reduction of LA into GVL, efficient electrode materials can be hopefully designed by this strategy, which has not yet been reported.

In another aspect, electrolytes play a crucial role on the efficiency of electrocatalysis.^24^,^25^ Recently, ionic liquids (ILs) have been considered as excellent alternatives for conventional electrolytes in electrochemistry due to their wide electrochemical window, high ionic conductivity, and structural designability.^26^ As efficient electrolytes, ILs have been proved to improve the electrochemical reduction of CO_2_ with high faradaic efficiencies and product selectivities.^27^ Therefore, there is great potential to use ILs as electrolytes in electrochemical reduction of LA to enhance the faradaic efficiency and GVL selectivity, which has gained no concern.

Development of highly efficient and selective route for electrochemical reduction of LA into GVL using water as the hydrogen source is highly desired. Herein, we report electrochemical reduction of LA into GVL using partially oxidized PbS as the electrocatalysts in a ternary electrolyte consisting of an IL, H_2_O and acetonitrile (MeCN). Interestingly, the combination of partially oxidized PbS electrocatalysts and the IL-containing electrolyte provided an unprecedented faradaic efficiency (78.6%) of GVL with a current density (13.5 mA cm^–2^). Meanwhile, GVL was the only product from LA reduction, indicating the outstanding selectivity of this proposed electrocatalytic systems.

## Results and discussion

### Synthesis and characterization of PbS-based electrocatalysts

**Fig. 1:**
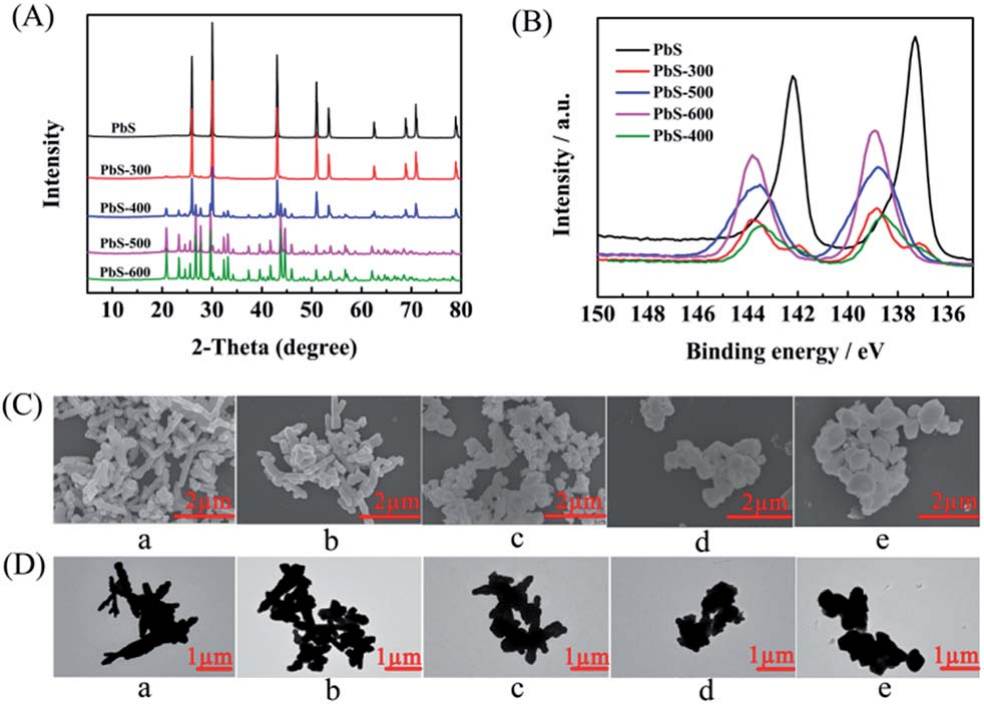
Characterization of the as-prepared PbS-based materials. (A) XRD, (B) XPS of Pb 4f, (C) SEM images, and (D) TEM images. (a, b, c, d, and e represent the PbS, PbS-300, PbS-400, PbS-500, and PbS-600).

The PbS-based materials (denoted as PbS-*X*, and *X* represents the calcination temperature) were prepared in a deep eutectic solvent consisting of thiourea and PEG-200 by a simple solvothermal reaction and subsequent calcination at different temperatures, and the detailed procedures were discussed in Experimental section. The synthesized materials were characterized by various techniques. X-ray diffraction (XRD) examinations indicated that the obtained material without calcination had the characteristic peak of PbS (JCPDS: 05-0592), suggesting the formation of PbS directly through the solvothermal route. As described in the XRD patterns (Fig. 1A), the phase of PbSO_4_ (JCPDS: 36-1461) was formed on the surface when the PbS was calcined at above 300 °C, and the amount of PbSO_4_ increased with the increasing calcination temperature. Meanwhile, X-ray photoelectron spectra (XPS) showed that the peak intensity for Pb^2+^ in PbS^28^ decreased while that in PbSO_4_ (ref. 29) increased with the increasing calcination temperature (Fig. 1B), which was consistent with the results from XRD. When the temperature was above 400 °C, the peak of PbS disappeared, indicating the surface of PbS was completely transformed into PbSO_4_. Additionally, the peak for S^6+^ in PbSO_4_ (ref. 30 and 31) appeared after calcination (Fig. S1†), further confirming the formation of PbSO_4_. The scanning electron microscopy (SEM, Fig. 1C) and transmission electron microscopy (TEM, Fig. 1D) images clearly demonstrated the morphology change of the obtained five materials calcined at different temperatures. The elements mapping from SEM indicated that Pb, S and O were uniformly dispersed (Fig. S2–S6†), respectively. The chemical composition of each material was obtained by ICP-AES analysis (Table S1†), which was consisted with the results from XRD and XPS examinations.

### Performance of various electrocatalysts

**Fig. 2:**
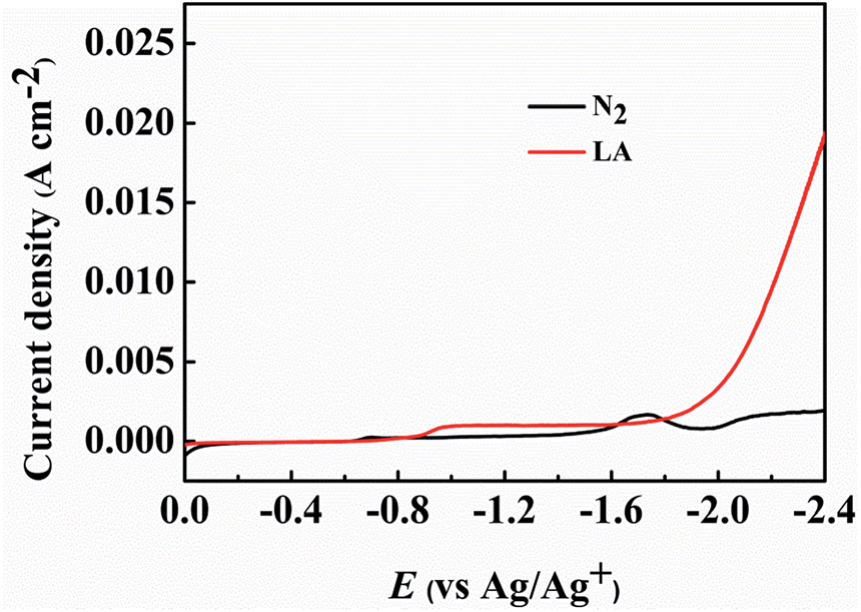
LSV curves using PbS-400/CP electrode in [Bmim]BF_4_ (1.8 wt%)–MeCN–H_2_O (1.8 wt%) electrolyte.

The obtained PbS-based materials were spread onto carbon paper (CP) as the electrodes for electrochemical reduction of LA (denoted as PbS-*X*/CP). To examine the feasibility of the prepared PbS-*X*/CP electrodes for LA electrocatalytic reduction, linear sweep voltammetry (LSV) was initially conducted in a typical H-type cell containing three electrodes using [Bmim]BF_4_–MeCN–H_2_O as catholyte and H_2_SO_4_ (0.5 M) as anolyte, and the applied potential was swept from 0 to –2.4 V *vs.* Ag/Ag^+^ with a scan rate of 20 mV s^–1^. As showed in Fig. 2 and S7,† the current density was obviously enhanced when LA was added into the electrolyte in comparison to that without LA, indicating the occurrence of LA electrochemical reduction on the PbS-*X*/CP electrode. More importantly, PbS-400/CP showed much better performance for the electrochemical reduction of LA to GVL in [Bmim]BF_4_ (1.8 wt%)–MeCN–H_2_O (1.8 wt%) electrolyte than other electrodes (Table 1). In addition, when directly using CP as the electrode, the faradaic efficiency of GVL was very low (<2%), indicating the catalytic role of the PbS-based catalysts for LA reduction. The results above suggested that PbS-400/CP could be used as a superior electrode material for LA electroreduction to GVL.

**Table 1:** The faradaic efficiency and current density of the electroreduction of LA to GVL using different electrodes at an applied potential of –2.15 V (*vs.* Ag/Ag^+^) in [Bmim]BF_4_ (1.8 wt%)–MeCN–H_2_O (1.8 wt%) catholyte for 4 h

Entry	Electrode	FE_GVL_^*a*^ (%)	*J* ^*b*^ (mA cm^–2^)
1	PbS/CP	15.6	17.5
2	PbS-300/CP	17.4	16.9
3	PbS-400/CP	78.6	13.5
4	PbS-500/CP	41.5	16.2
5	PbS-600/CP	46.4	15.6

^*a*^FE_GVL_ is the faradaic efficiency of GVL.

^*b*^ *J* represents the total current density.

As shown in Tables 1 and S1,† the degree of PbS oxidation to PbSO_4_ significantly affected the performance of the synthesized PbS-based electrode. In order to get some evidence to explain this phenomenon, some characterizations were carried out. First, the electrochemical impedance spectroscopy (EIS) of the synthesized PbS-based materials was determined (Fig. S8–S12†) to obtain detailed insight into the interfacial information of electrode/electrolyte, and the high and medium frequency data was fitted using a simple equivalent circuit (Fig. S13†). The charge transfer resistances (*R*_ct_) were 41.75, 45.20, 38.04, 43.92, and 41.07 Ω cm^–2^ for PbS/CP, PbS-300/CP, PbS-400/CP, PbS-500/CP, and PbS-600/CP, respectively. PbS-400/CP showed the lowest *R*_ct_, demonstrating easier electron transfer on the interface of PbS-400/CP and the catholyte, which was beneficial for the electrochemical reduction of LA. Meanwhile, the performance of PbS-400/CP was much better than those of PbS/CP (no oxidation), PbS-300/CP (lower degree of PbS oxidation than PbS-400/CP), PbS-500/CP (higher degree of PbS oxidation than PbS-400/CP), and PbS-600/CP (complete oxidation), verifying that suitable degree of PbS oxidation to PbSO_4_ affected the electrocatalytic performance significantly, and PbS and the formed PbSO_4_ showed a synergistic effect to enhance the reaction performance. Second, as shown in the SEM and TEM images (Fig. 1C and D), partial oxidation resulted in the morphology change of the materials. Looser structure of PbS-400, PbS-500 and PbS-600 (Fig. 1C) was generated, and thus more active sites of the catalysts were exposed, which could enhance the reaction. On the basis of the above discussion, we can conclude that the suitable degree of PbS oxidation to PbSO_4_ in PbS-400/CP is favorable to improving the catalytic performance, and PbS-400/CP yielded the best results.

### Influence of different ILs

ILs played a significant role in the electrochemical reduction of LA. Control experiment indicated that no LA was reduced in the absence of any ILs. Among the examined ILs, [Bmim]BF_4_ (Table 2, entry 2) showed higher faradaic efficiency of GVL over PbS-400/CP electrode. For imidazolium-based ILs, the side chain on the imidazole ring significantly affected the electrochemical reduction of LA. Although [Emim]BF_4_ showed the highest current density and conductivity (Table 2, entry 1), the faradaic efficiency for GVL was much lower than that in [Bmim]BF_4_ because higher current density and conductivity could also enhance the hydrogen evolution. Moreover, the steric effect increased with increasing length of the side chain, which could weaken the interaction between imidazole ring and carbonyl group in LA (a key step to activate the LA for electrochemical reduction). Therefore, the faradaic efficiency for GVL decreased when the chain in the ILs was too long, as shown by the results using [Hmim]BF_4_ and [Omim]BF_4_ (Table 2, entries 3 and 4). Additionally, imidazolium-based ILs had better performance than the quaternary ammonium- and phosphonium-based ILs (Table 2, entries 5 and 6), mainly because that the interaction between the carbonyl group in LA and the two ILs was very week (Fig. S14†) and their conductivities were lower than those of imidazolium-based ILs. The results above demonstrated that [Bmim]BF_4_ was the optimal IL in our catalytic system.

**Table 2:** The faradaic efficiency and current density of the electroreduction of LA to GVL using PbS-400/CP electrode at an applied potential of –2.15 V (*vs.* Ag/Ag^+^) in (1.8 wt%)–MeCN–H_2_O (1.8 wt%) catholyte for 4 h

Entry	IL	FE_GVL_^*a*^ (%)	*J* ^*b*^ (mA cm^–2^)	Conductivity (ms cm^–1^)
1	[Emim]BF_4_	12.4	33.9	9.43
2	[Bmim]BF_4_	78.6	13.5	7.95
3	[Hmim]BF_4_	17.2	10.7	7.02
4	[Omim]BF_4_	14.3	9.1	6.32
5	[TBA]BF_4_	6.8	9.6	5.61
6	[TBP]BF_4_	9.3	8.0	4.74

^*a*^FE_GVL_ is the faradaic efficiency of GVL.

^*b*^ *J* represents the total current density.

### Effect of various reaction parameters

**Fig. 3:**
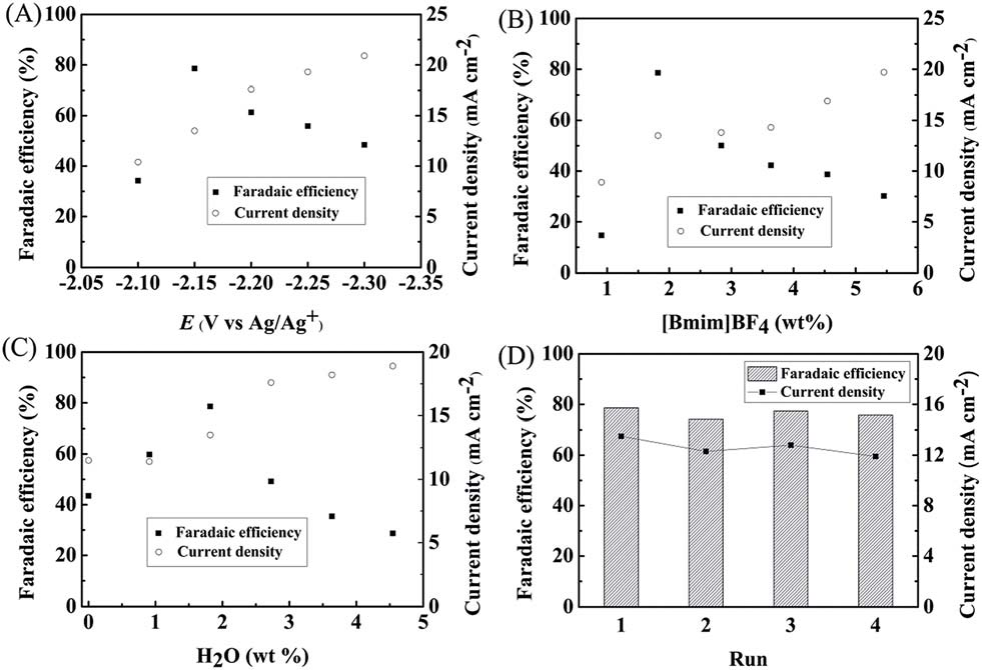
Effects of various parameters on electrochemical reduction of LA over PbS-400/CP electrode in the ternary electrolyte of [Bmim]BF_4_–MeCN–H_2_O for 4 h. (A) Applied potential, (B) [Bmim]BF_4_ concentration, (C) H_2_O amount, and (D) the reusability of PbS-400/CP electrode for the electrochemical reduction of LA in [Bmim]BF_4_ (1.8 wt%)–MeCN–H_2_O (1.8 wt%) at –2.15 V *vs.* Ag/Ag^+^ for 4 h.

We studied the effect of applied potential on the efficiency of the electrochemical reduction of LA into GVL over PbS-400/CP electrode. As illustrated in Fig. 3A, the current density increased with increasing applied potential. At –2.15 V *vs.* Ag/Ag^+^, the faradaic efficiency of GVL could reach 78.6% with a current density of 13.5 mA cm^–2^. At lower potentials, the faradaic efficiency of LA was lower and competitive hydrogen evolution from H_2_O electrolysis was the main side reaction. At higher potentials, migration rate of H^+^ was faster than LA and more H^+^ was reduced. Therefore, –2.15 V *vs.* Ag/Ag^+^ was selected as the optimal potential for subsequent experiments.

We further determined the effect of composition of the electrolyte on the reaction. As can be known from Fig. 3B, 1.8 wt% [Bmim]BF_4_ in the electrolyte yielded maximum faradaic efficiency of GVL (78.6%) at an applied potential of –2.15 V *vs.* Ag/Ag^+^. In the reaction process, IL could promote the electrochemical reduction of LA by activating the carbonyl group in LA through the formation of hydrogen bonding (Fig. S14†). Moreover, the number of ionic species in the electrolyte increased with the increase of IL concentration, which could increase the conductivity of the electrolyte. However, higher IL concentration could also increase hydrogen evolution with the increasing conductivity. These two opposite effects resulted in the best result at 1.8 wt% of [Bmim]BF_4_. In another aspect, the amount of H_2_O in the electrolyte could also affect the reduction of LA (Fig. 3C). A certain amount of H_2_O could increase the dissociation of the IL, resulting in an increasing amount of ionic species in the electrolyte, which was beneficial for LA reduction. However, as a protic solvent, H_2_O could provide high proton concentration to enhance the hydrogen evolution,^32^ which would lower the faradaic efficiency of GVL. Therefore, 1.8 wt% of H_2_O was the suitable concentration in our reaction systems (Fig. 3C). In addition, the PbS-400/CP electrode could be reused at least four times without obvious decrease in faradaic efficiency and current density for the electrochemical reduction of LA to GVL (Fig. 3D). Meanwhile, the difference in the XRD patterns of the virgin and used catalysts was not notable (Fig. S15†), further indicating the stability of the PbS-400/CP electrode in the reaction.

### Reaction pathway and mechanism

**Scheme 1:**
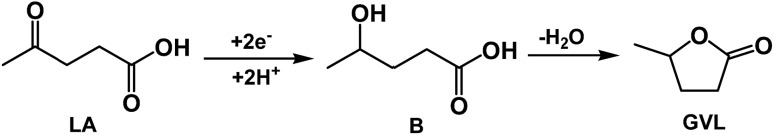
Reaction pathway for electrochemical reduction of LA into GVL over PbS-400/CP electrode in [Bmim]BF_4_-containing catholyte.

**Fig. 4:**
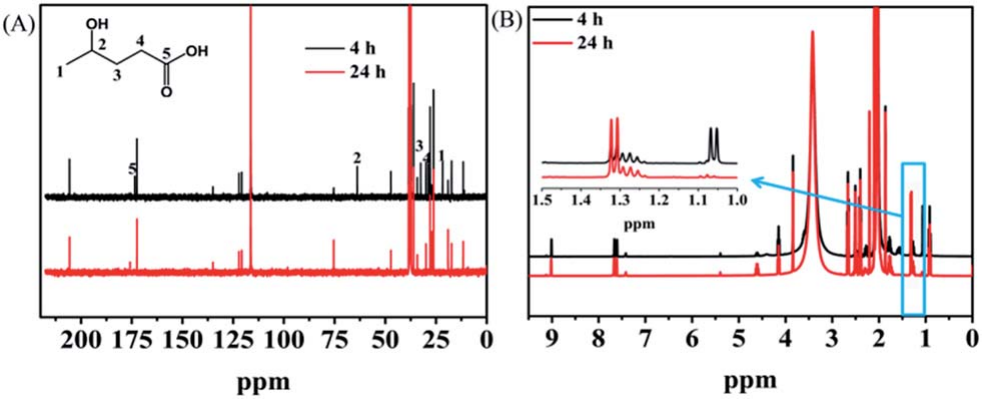
NMR analysis of the electrolyte after electrolysis for electrochemical reduction of LA over PbS-400/CP in [Bmim]BF_4_ (1.8 wt%)–MeCN–H_2_O (1.8 wt%) at –2.15 V *vs.* Ag/Ag^+^. (A) ^13^C NMR and (B) ^1^H NMR spectra. The black line represented the NMR result determined immediately after the reaction (4 h), and the red line is the result obtained after the electrolyte was kept static for 24 h.

Investigation of the reaction pathway is very interesting. Generally, conversion of LA into GVL was a two-step reaction, including the hydrogenation of the carbonyl group in LA to form the intermediate 4-hydroxy pentanoic acid (B), and the intramolecular esterification of B to generate the desired GVL (Scheme 1). In the reaction process, the intermediate B that could be clearly detected through ^13^C NMR (Fig. 4A) and MS (Fig. S16†) was formed by obtaining two electrons and two protons through electrocatalysis. Furthermore, the faradaic efficiency of GVL still increased even when the electroreaction was stopped for 24 h. Through ^1^H NMR analysis (Fig. 4B), we found that the characteristic double peaks at 1.05 ppm (belonged to –CH_3_ in B) disappeared after 24 h, while the intensity of the peak appeared at 1.31 ppm (belonged to –CH_3_ in GVL) increased. Meanwhile, in ^13^C NMR (Fig. 4A), the signal of carbon for B disappeared after 24 h. These results indicated that the intramolecular esterification of B might not be an electrocatalytic process. Additionally, a control experiment using pyruvic acid as the substrate (Scheme S1†) showed that lactic acid was the only product as examined by GC-MS (Fig. S17†), indicating that the carbonyl group (C=O) was the only reduced group in our electrochemical system.

**Scheme 2:**
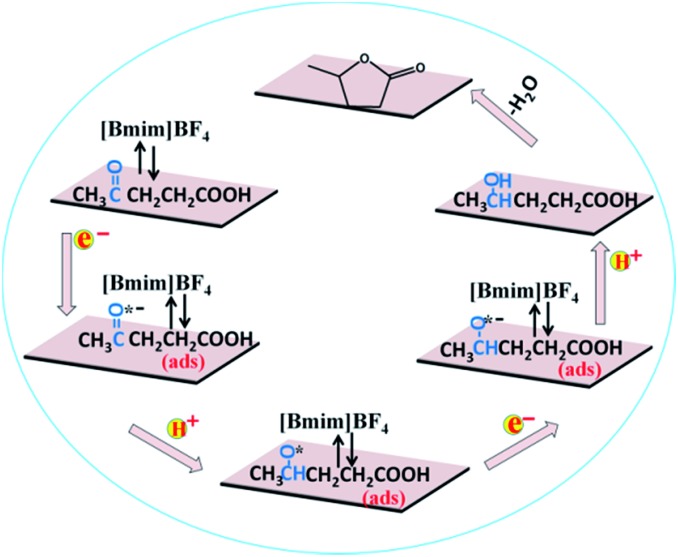
Possible reaction mechanism for electrochemical reduction of LA to GVL over PbS-400/CP electrode in [Bmim]BF_4_-containing catholyte.

Based on the above experimental results, a speculated mechanism was proposed for the electrochemical reduction of LA into GVL over PbS-400/CP electrode in [Bmim]BF_4_–MeCN–H_2_O electrolyte (Scheme 2). First, the carbonyl group (C=O) was activated by the formation of a complex [Bmim–LA]^+^ through the hydrogen bonding interaction between LA and the [Bmim]^+^ cation in IL, which was beneficial for the subsequent electroreduction of C=O groups in LA. Then, the generated complex absorbed on the surface of PbS-400/CP electrode to form –CO˙^–^ (ads) intermediate by obtaining one electron. After that, the –CO˙^–^ (ads) received one proton to form –CHO˙ (ads), which would be converted into –CHOH (intermediate B) by obtaining one electron and one proton. Finally, GVL was generated by intramolecular esterification.

## Conclusions

In conclusion, PbS-based electrocatalysts were synthesized for electrocatalytic reduction of LA to GVL using water as the hydrogen source. The performance of the catalysts could be easily tuned by the degree of partial oxidation of the PbS surface to PbSO_4_, which could be controlled by the calcination temperature. The PbS-400/CP showed the best performance in a ternary electrolytes consisting of ionic liquid, organic solvent, and H_2_O. The faradaic efficiency of GVL over PbS-400/CP could reach 78.6% with a current density of 13.5 mA cm^–2^, and GVL was the only product from LA reduction. Mechanism analysis showed that LA was converted into GVL through electrochemical hydrogenation followed by subsequent intramolecular esterification. We believe that combination of metal chalcogenides and ionic liquid-based electrolytes opens a promising avenue for electrochemical transformation of biomass with high efficiency and selectivity, and partial change of surface composition will be an attractive strategy for designing efficient electrocatalysts.

## Experimental

### Materials

Ionic liquids, including 1-ethyl-3-methylimidazolium tetrafluoroborate ([Emim]BF_4_, >99%), 1-butyl-3-methylimidazolium tetrafluoroborate ([Bmim]BF_4_, >99%), 1-octyl-3-methylimidazolium tetrafluoroborate ([Omim]BF_4_, >99%), and 1-hexyl-3-methylimidazolium tetrafluoroborate ([Hmim]BF_4_, >99%) were purchased from the Centre of Green Chemistry and Catalysis, Lanzhou Institute of Chemical Physics, Chinese Academy of Sciences. Polyethylene glycol (PEG-200, >99%), thiourea (>99%), lead(ii) acetate trihydrate (>99%), tetrabutylammonium tetrafluoroborate ([TBA]BF_4_, >99%), and tetrabutylphosphonium tetrafluoroborate ([TPA]BF_4_, >99%) were supplied by J&K Scientific Co., Ltd. Nafion D-521 dispersion (5% w/w in water and 1-propanol, ≥0.92 meg g^–1^ exchange capacity), Nafion N-117 membrane (0.180 mm thick, ≥0.90 meg g^–1^ exchange capacity), and Toray Carbon Paper (CP, TGP-H-60, 19 × 19 cm) were purchased from Alfa Aesar China Co., Ltd.

### Preparation of lead sulfide (PbS)

Firstly, a deep eutectic solvent consisting of PEG-200 and thiourea were prepared with the mole ratio of 2 : 1 following the method reported.^33^ Then, 3.0 g lead(ii) acetate trihydrate was dissolved into the deep eutectic solvent (70.0 g) with the assistance of ultrasound. The above mixture was moved into a stainless steel reactor with a Teflon coating, and the reactor was heated at 180 °C for 24 h. After reaction, the product, namely, lead sulfide (PbS) was washed by water and ethanol three times, and was dried in vacuum at 40 °C for 24 h.

### Preparation of electrocatalysts

The as-prepared PbS was calcined in muffle furnace at desired temperature (300, 400, 500, and 600 °C) in the air for 12 h. The product was denoted as PbS-*X* (*X* represents the calcination temperature). Then, 8 mg PbS-*X* (300, 400, 500, and 600) was suspended in 1 mL ethanol with 20 μL Nafion D-521 dispersion (5 wt%) to form a homogeneous ink with the aid of ultrasound. Then, the ink was spread onto the carbon paper (CP) surface (1 cm × 1 cm) by a micropipette and then dried under room temperature. These electrodes were denoted as PbS-*X*/CP.

### Characterization

The scanning electron microscopy (SEM) measurements and element mappings were performed on a Hitachi S-4800 scanning electron microscope operated at 15 kV. The transmission electron microscopy (TEM) images were obtained using a TEM JEOL-1011 with an accelerating voltage of 120 kV. Powder X-ray diffraction (XRD) patterns were collected on a Rigaku D/max-2500 X-ray diffractometer using Cu Kα radiation (*λ* = 0.154 nm). X-Ray photoelectron spectroscopy (XPS) measurements were carried out on a ESCAL Lab 220i-XL spectrometer. The content of Pb and S in the as-prepared PbS materials were determined by inductively coupled plasma atomic emission spectroscopy (ICP-AES VISTA-MPX). ^1^H NMR and ^13^C NMR spectra were recorded on a Bruker Avance III HD 400 MHz NMR spectrometer.

### Linear sweep voltammetry (LSV) measurement

An electrochemical workstation (CHI 660E, Shanghai CH Instruments Co., China) was used in all the experiments. Linear sweep voltammetry (LSV) measurements were conducted in an H-type cell separated by Nafion membrane. The system had three electrodes including working electrode (PbS-*X*/CP), a platinum gauze auxiliary electrode, and an Ag/Ag^+^ (0.01 M AgNO_3_ in 0.1 M TBAP–MeCN) reference electrode. Prior to experiment, electrolyte was bubbled with N_2_ for 30 min so that the air was removed completely. The LSV measurement was carried out in the potential range of 0 to –2.4 V *vs.* Ag/Ag^+^ at a sweep rate of 20 mV s^–1^. The above process was under the slight magnetic stirring.

### Electrochemical impedance spectroscopy (EIS) study

The EIS experiment was carried out in a single compartment cell with three-electrode configuration consisting of working electrode, a platinum gauze auxiliary electrode, and Ag/Ag^+^ (0.01 M AgNO_3_ in 0.1 M TBAP–MeCN) reference electrode. The impedance spectra were recorded using potentiostatic mode at an open circuit potential of 100 kHz to 100 mHz with an amplitude of 5 mV. The data obtained from the EIS measurements were fitted by the ZSimpwin software.

### Electrolysis of LA and product analysis

Electrochemical reduction of LA was conducted at room temperature in a typical H-type cell, which was similar with that used for electrochemical reduction of CO_2_.^27^ Namely, IL-based electrolytes and H_2_SO_4_ aqueous solution (0.5 M) were used as cathodic and anodic electrolytes, respectively. The amount of electrolyte in each chamber was 5.5 g in all experiments. Prior to electrolysis, N_2_ was bubbled through the catholyte for 30 min with stirring, then LA (200 μL) was added into the catholyte. The liquid product was analyzed by ^1^H NMR (Bruker Avance III 400 HD spectrometer) in DMSO-d_6_ with TMS as an internal standard. The gaseous product was analyzed by gas chromatography (GC, HP 4890D) equipped with TCD detector using helium as the internal standard. The faradaic efficiency and current density of the products were calculated on the basis of GC and NMR analysis.

### Calculation of faradaic efficiency for GVL

In the reaction process, 2 moles of electrons were consumed to produce 1 mole of GVL. Therefore, the faradaic efficiency of GVL could be obtained on the basis of the following formula.
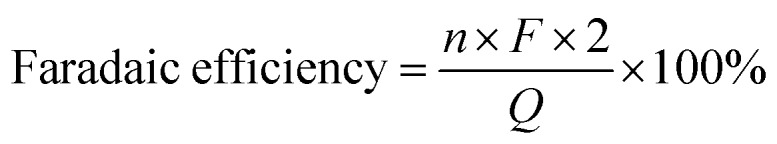
 *Q*, *F*, and *n* represented the total quantity of consumed electricity, constant of Faraday, and mole of GVL generated, respectively.

## Conflicts of interest

There are no conflicts to declare.

## Supplementary Material

Supplementary information

## References

[cit1] Zeng H., Cao D., Qiu Z., Li C.-J. (2018). Angew. Chem., Int. Ed..

[cit2] Reichert E., Wintringer R., Volmer D. A., Hempelmann R. (2012). Phys. Chem. Chem. Phys..

[cit3] Chadderdon X. H., Chadderdon D. J., Matthiesen J. E., Qiu Y., Carraher J. M., Tessonnier J.-P., Li W. (2017). J. Am. Chem. Soc..

[cit4] Xia Q., Chen Z., Shao Y., Gong X., Wang H., Liu X., Parker S. F., Han X., Yang S., Wang Y. (2016). Nat. Commun..

[cit5] Wang M., Shi H., Camaioni D. M., Lercher J. A. (2017). Angew. Chem., Int. Ed..

[cit6] Ferlin F., Luciani L., Santoro S., Marrocchi A., Lanari D., Bechtoldt A., Ackermann L., Vaccaro L. (2018). Green Chem..

[cit7] Bechtoldt A., Baumert M. E., Vaccaro L., Ackermann L. (2018). Green Chem..

[cit8] Luo W., Sankar M., Beale A. M., He Q., Kiely C. J., Bruijnincx P. C. A., Weckhuysen B. M. (2015). Nat. Commun..

[cit9] Yu I. K. M., Tsang D. C. W., Yip A. C. K., Hunt A. J., Sherwood J., Shang J., Song H., Ok Y. S., Poon C. S. (2018). Green Chem..

[cit10] Yi Y., Liu H., Xiao L.-P., Wang B., Song G. (2018). ChemSusChem.

[cit11] Song J., Zhou B., Zhou H., Wu L., Meng Q., Liu Z., Han B. (2015). Angew. Chem., Int. Ed..

[cit12] Albani D., Li Q., Vilé G., Mitchell S., Almora-Barrios N., Witte P. T., López N., Pérez-Ramírez J. (2017). Green Chem..

[cit13] Ftouni J., Genuino H. C., Muñoz-Murillo A., Bruijnincx P. C. A., Weckhuysen B. M. (2017). ChemSusChem.

[cit14] Xin L., Zhang Z., Qi J., Chadderdon D. J., Qiu Y., Warsko K. M., Li W. (2013). ChemSusChem.

[cit15] Santos T. R., Nilges P., Sauter W., Harnisch F., Schröder U. (2015). RSC Adv..

[cit16] Hesari M., Swanick K. N., Lu J.-S., Whyte R., Wang S., Ding Z. (2015). J. Am. Chem. Soc..

[cit17] Zhang X., Lai Z., Tan C., Zhang H. (2016). Angew. Chem., Int. Ed..

[cit18] Luo Z., Ouyang Y., Zhang H., Xiao M., Ge J., Jiang Z., Wang J., Tang D., Cao X., Liu C., Xing W. (2018). Nat. Commun..

[cit19] Siao M. D., Shen W. C., Chen R. S., Chang Z. W., Shih M. C., Chiu Y. P., Cheng C.-M. (2018). Nat. Commun..

[cit20] Sun X., Deng H., Zhu W., Yu Z., Wu C., Xie Y. (2016). Angew. Chem., Int. Ed..

[cit21] Xie J., Zhang J., Li S., Grote F., Zhang X., Zhang H., Wang R., Lei Y., Pan B., Xie Y. (2013). J. Am. Chem. Soc..

[cit22] Jeong S., Yoo D., Jang J., Kim M., Cheon J. (2012). J. Am. Chem. Soc..

[cit23] Chen J., Wu X.-J., Yin L., Li B., Hong X., Fan Z., Chen B., Xue C., Zhang H. (2015). Angew. Chem., Int. Ed..

[cit24] Zhu Q., Ma J., Kang X., Sun X., Liu H., Hu J., Liu Z., Han B. (2016). Angew. Chem., Int. Ed..

[cit25] Luo Z.-X., Xing Y.-Z., Ling Y.-C., Kleinhammes A., Wu Y. (2015). Nat. Commun..

[cit26] Gunawan C. A., Ge M., Zhao C. (2014). Nat. Commun..

[cit27] Sun X., Zhu Q., Kang X., Liu H., Qian Q., Zhang Z., Han B. (2016). Angew. Chem., Int. Ed..

[cit28] Li L., Dai H., Feng L., Luo D., Wang S., Sun X. (2015). Nanoscale Res. Lett..

[cit29] Xu J., Li X., Wang Z., Dong C. (2004). Chin. J. of Nonferrous Met..

[cit30] Wahlqvist M., Shchukarev A. (2007). J. Electron Spectrosc. Relat. Phenom..

[cit31] Ebitani K., Konno H., Tanaka T., Hattori H. (1992). J. Catal..

[cit32] Zhang L., Zhao Z., Gong J. (2017). Angew. Chem., Int. Ed..

[cit33] Jiang J., Yan C., Zhao X., Luo H., Xue Z., Mu T. (2017). Green Chem..

