# Supplementary material for: Surface engineering in PbS via partial oxidation: towards an advanced electrocatalyst for reduction of levulinic acid to γ-valerolactone
Source: Chem Sci. 2018 Dec 3;10(6):1754–9. doi: 10.1039/c8sc03161d (PMC6368243; doi:10.1039/c8sc03161d)
Supplement: Supplementary file 1 [file SC-010-C8SC03161D-s001.pdf]

Electronic Supplementary Material (ESI) for Chemical Science.
This journal is © The Royal Society of Chemistry 2018

Supporting information:

Surface engineering in PbS *via* partial oxidation: Towards an advanced electrocatalyst for reduction of levulinic acid to γ-valerolactone

Haoran Wu,[a,b] Jinliang Song,*[a] Chao Xie,[a,b] Yue Hu,[a,b] Pei Zhang,[a] Guanying Yang,[a] and Buxing Han*[a,b]

[a]Beijing National Laboratory for Molecular Science, CAS Key Laboratory of Colloid and Interface and Thermodynamics, CAS Research/Education Center for Excellence in Molecular Sciences, Institute of Chemistry, Chinese Academy of Sciences, Beijing 100190, China.

[b]School of Chemistry and Chemical Engineering, University of Chinese Academy of Sciences, Beijing 100049,China.

E-mails: songjl@iccas.ac.cn, hanbx@iccas.ac.cn

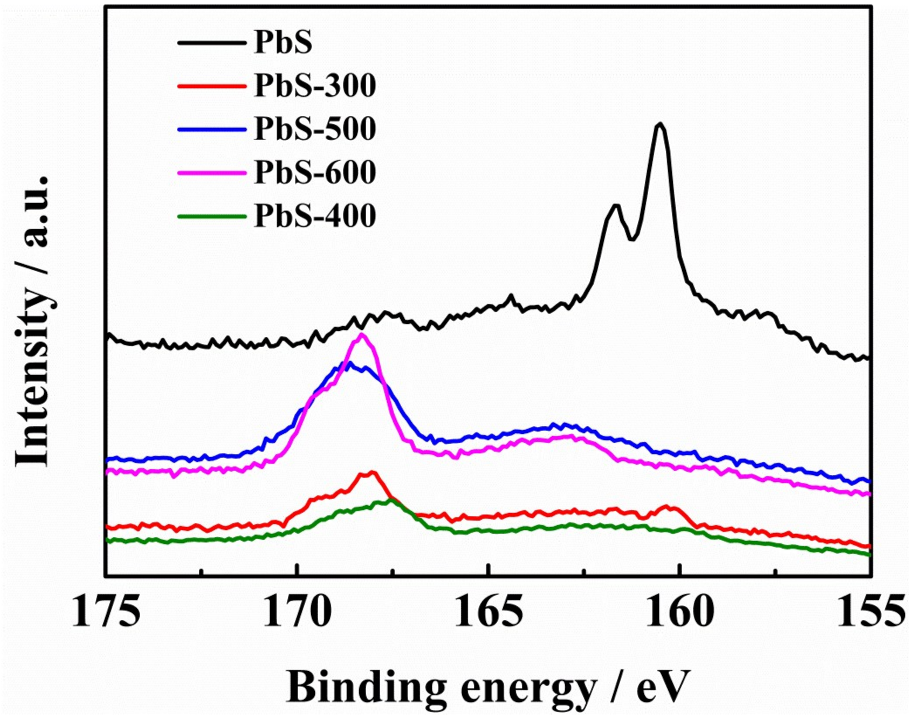

Fig. S1. XPS spectrum of S 2p in different PbS-X materials.

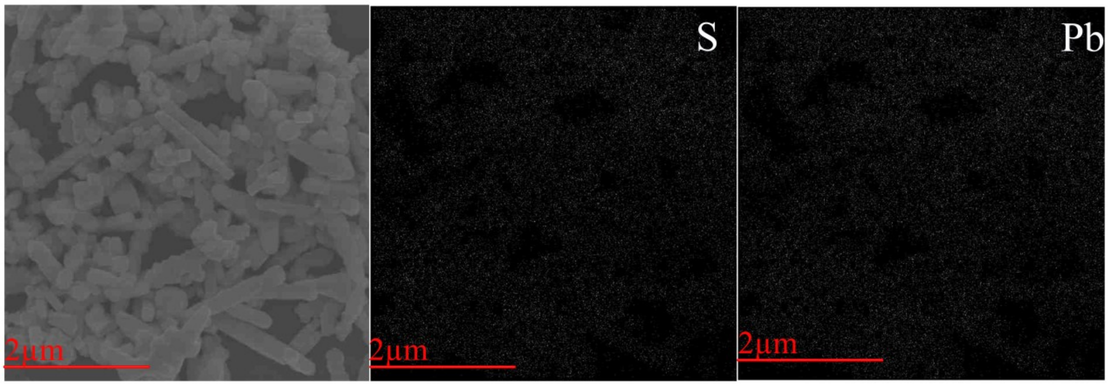

Fig. S2. Elements mapping of PbS.

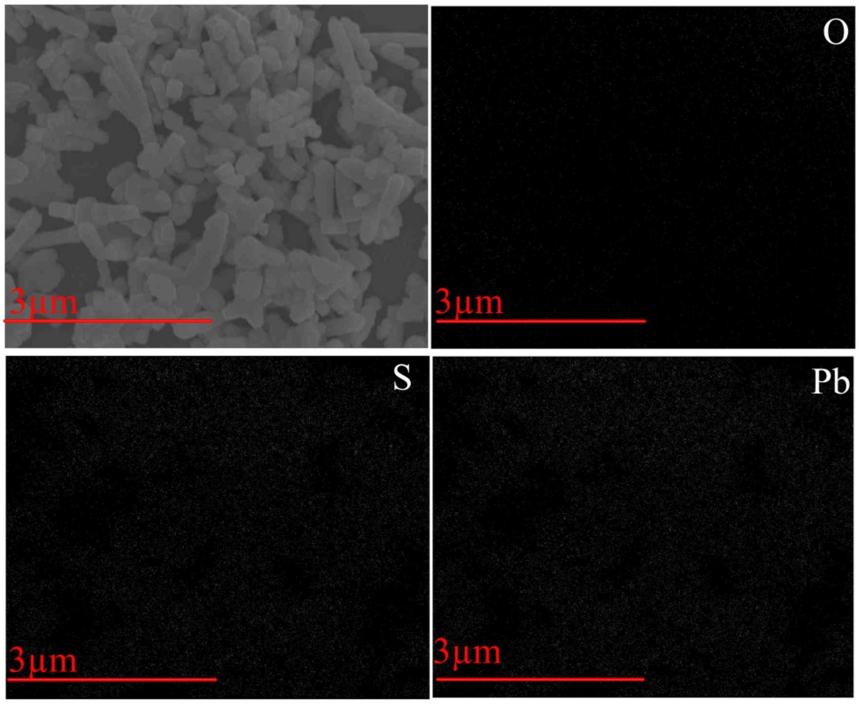

Fig. S3. Elements mapping of PbS-300.

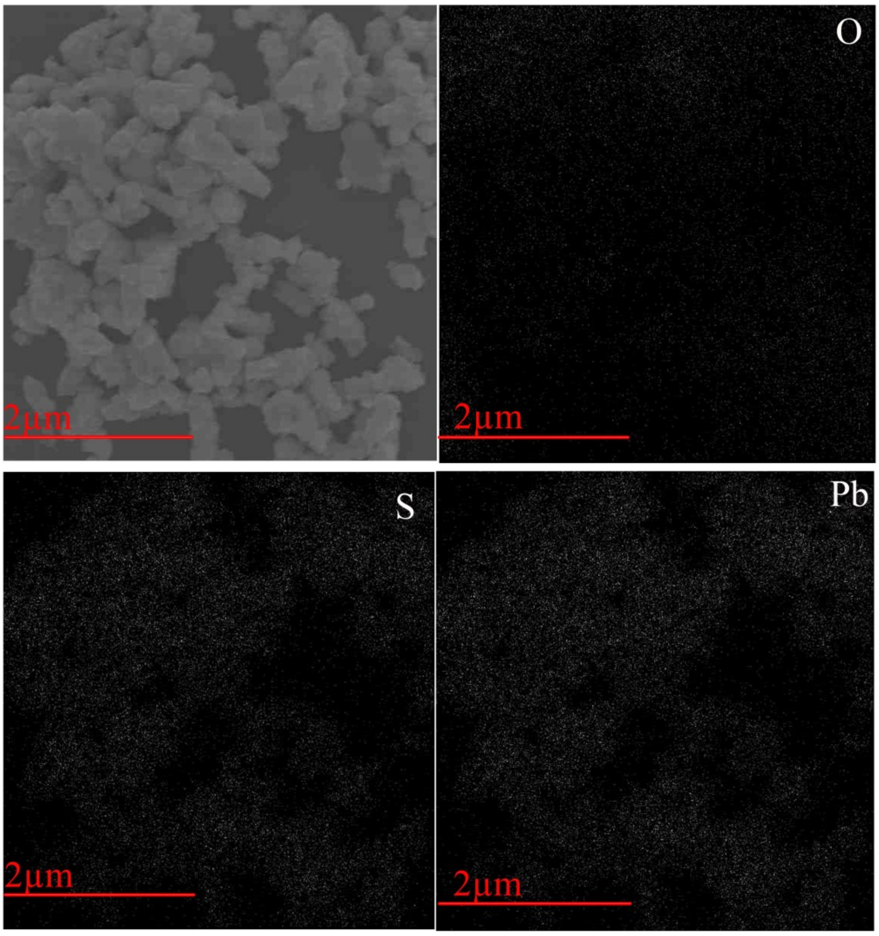

Fig. S4. Elements mapping of PbS-400.

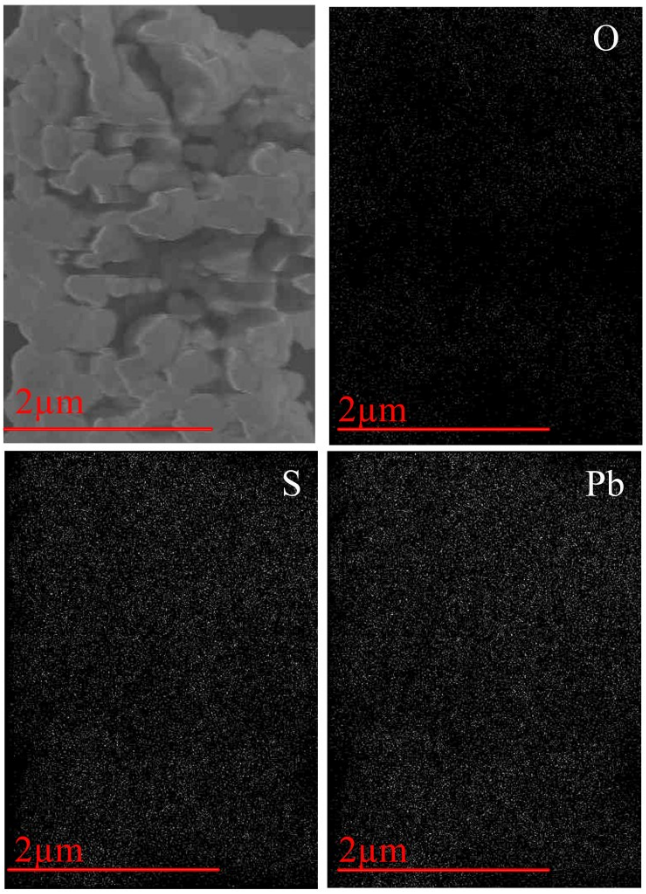

Fig. S5. Elements mapping of PbS-500.

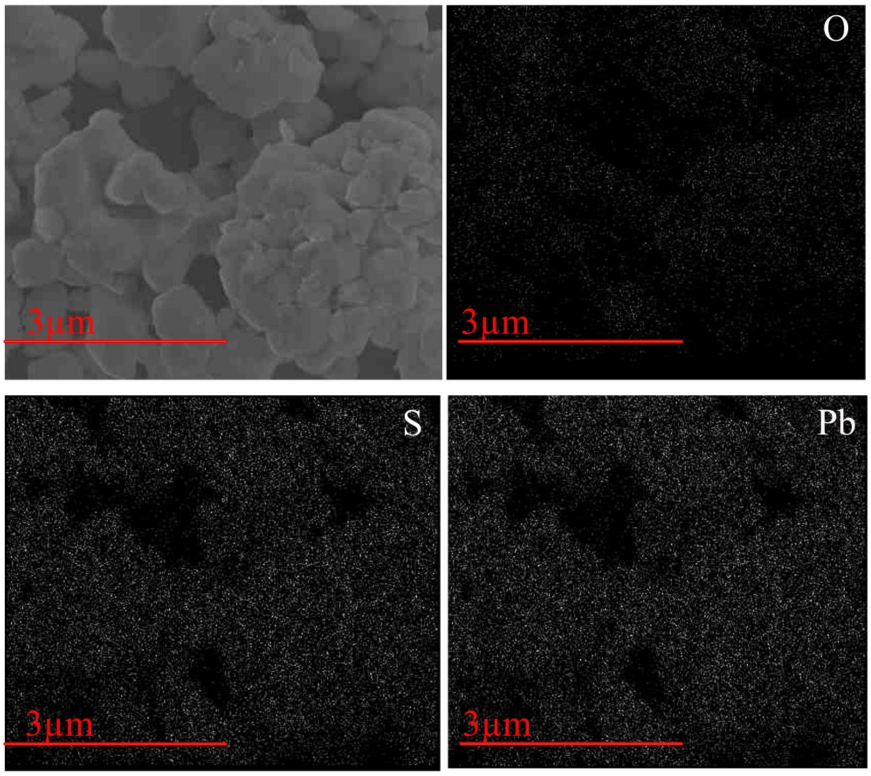

Fig. S6. Elements mapping of PbS-600.

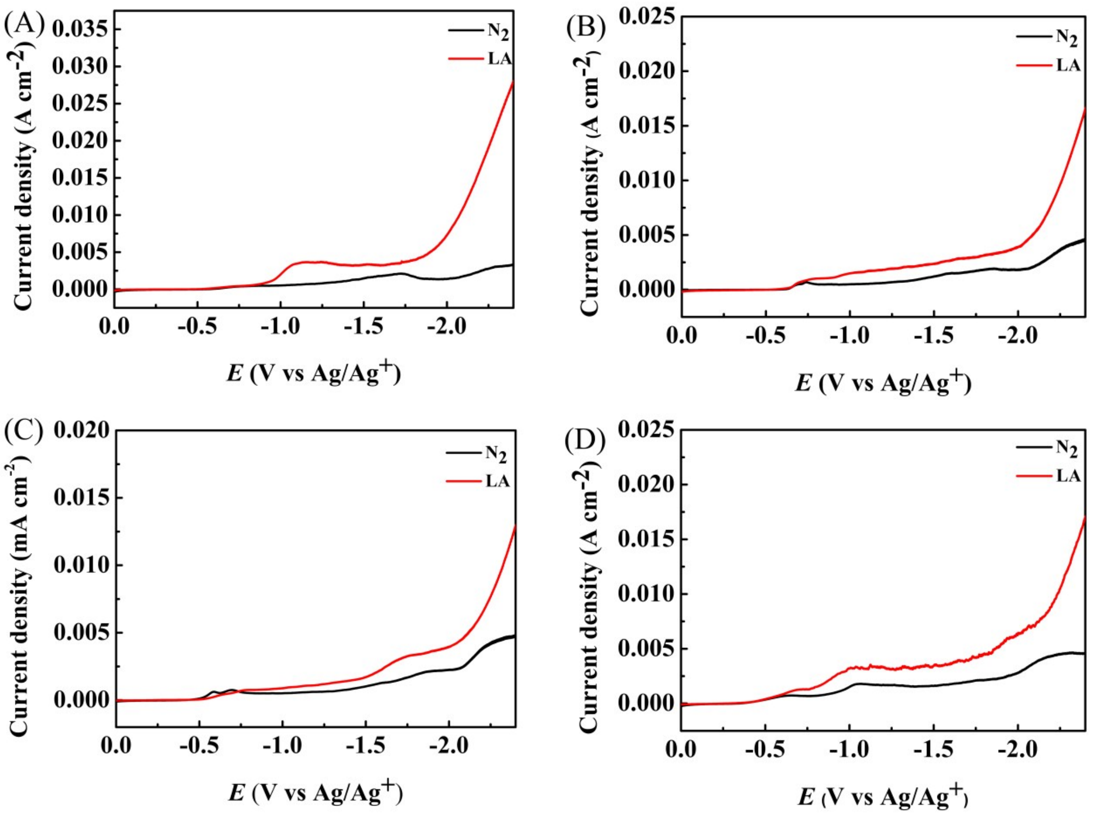

Fig. S7. LSV measurements using PbS-X/CP electrode for the electrochemical reduction of LA in the electrolyte of IL (1.8 wt%)-MeCN-H_2O (1.8 wt%) at room temperature. (A) PbS/CP, (B) PbS-300/CP, (C) PbS-500/CP, and (D) PbS-600/CP.

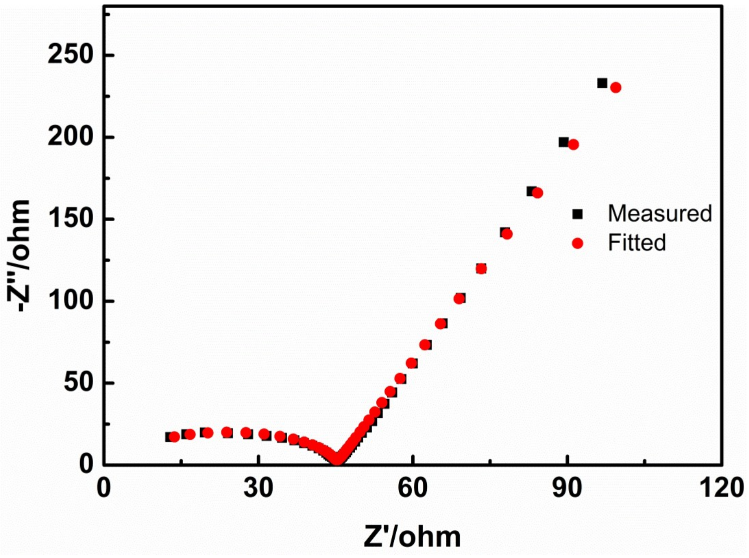

Fig. S8. The measured and fitted EIS spectra of PbS/CP in [Bmim]BF_4 (1.8 wt%)-MeCN-H_2O (1.8 wt%) electrolyte in the presence of LA.

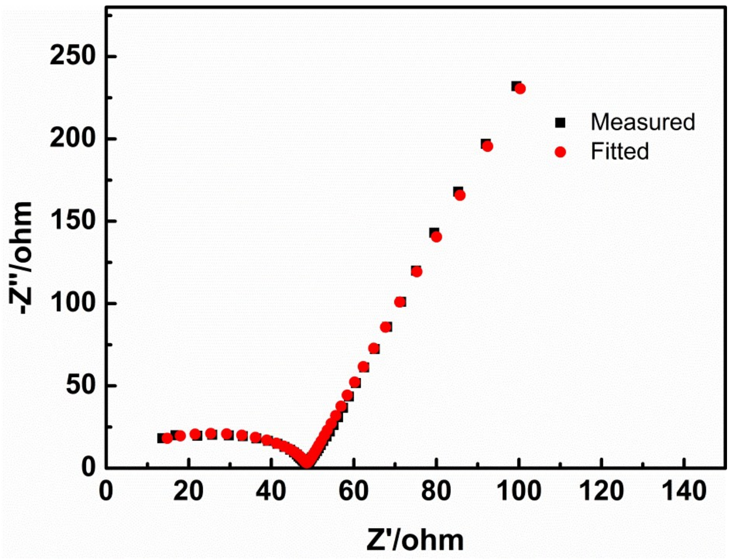

Fig. S9. The measured and fitted EIS spectra of PbS-300/CP in [Bmim]BF_4 (1.8 wt%)-MeCN-H_2O (1.8 wt%) electrolyte in the presence of LA.

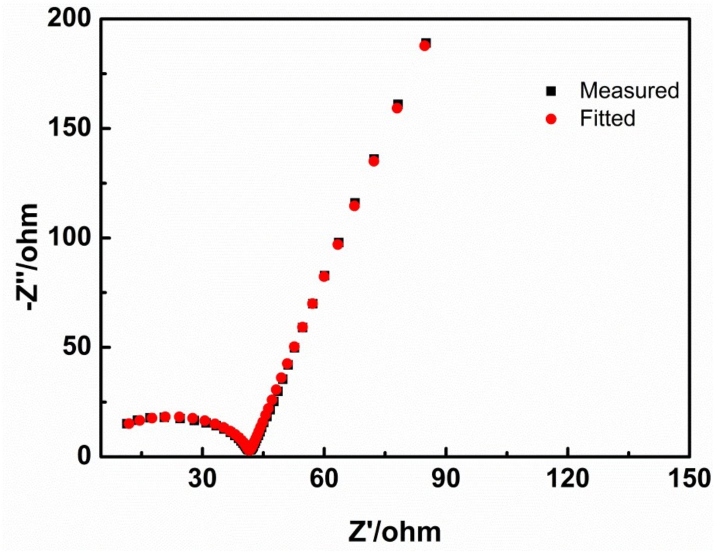

Fig. S10. The measured and fitted EIS spectra of PbS-400/CP in [Bmim]BF_4 (1.8 wt%)-MeCN-H_2O (1.8 wt%) electrolyte in the presence of LA.

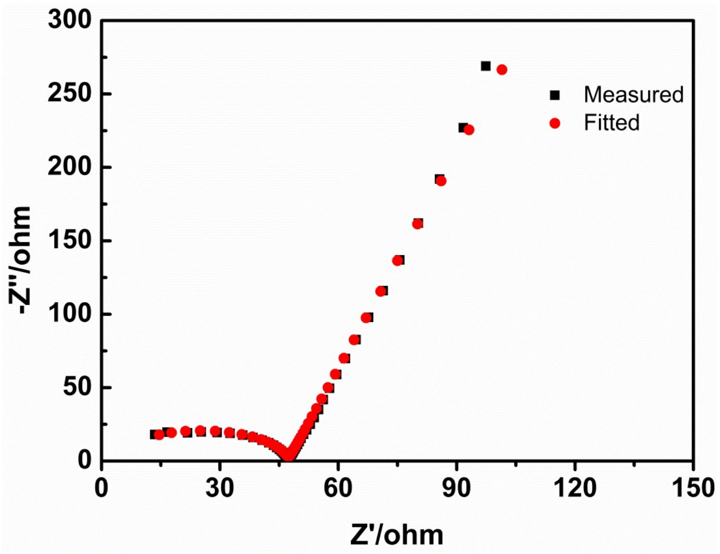

Fig. S11. The measured and fitted EIS spectra of PbS-500/CP in [Bmim]BF_4 (1.8 wt%)-MeCN-H_2O (1.8 wt%) electrolyte in the presence of LA.

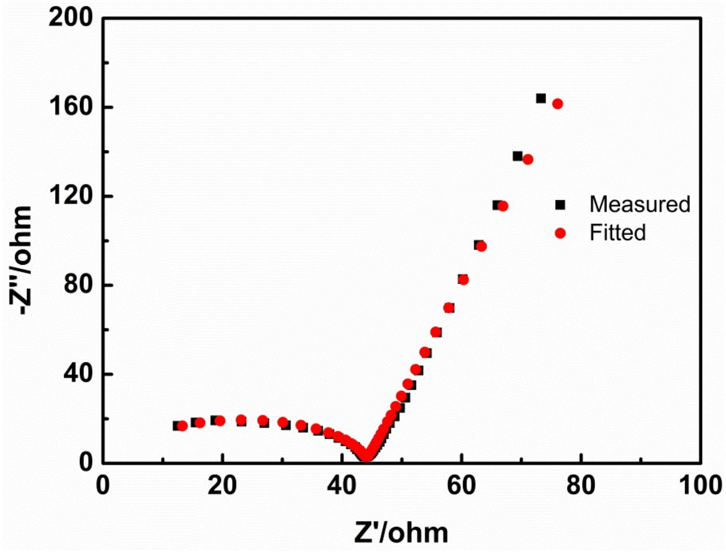

Fig. S12. The measured and fitted EIS spectra of PbS-600/CP in [Bmim]BF_4 (1.8 wt%)-MeCN-H_2O (1.8 wt%) electrolyte in the presence of LA.

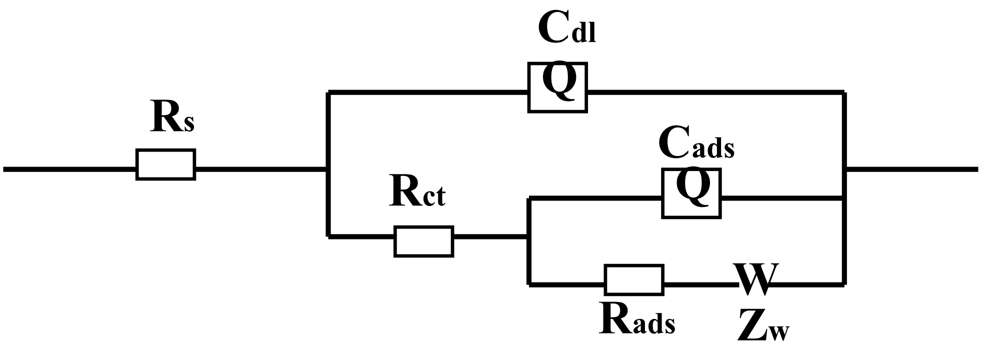

Fig. S13. Electrical equivalent circuit for fitting the measured impedance data. Rs, Cdl, Rct, Cads, Rads, and Zw represent solution resistance, double layer capacitance, electron transfer resistance, surface adsorption capacitance, surface adsorption resistance and Warburg impedance, respectively.

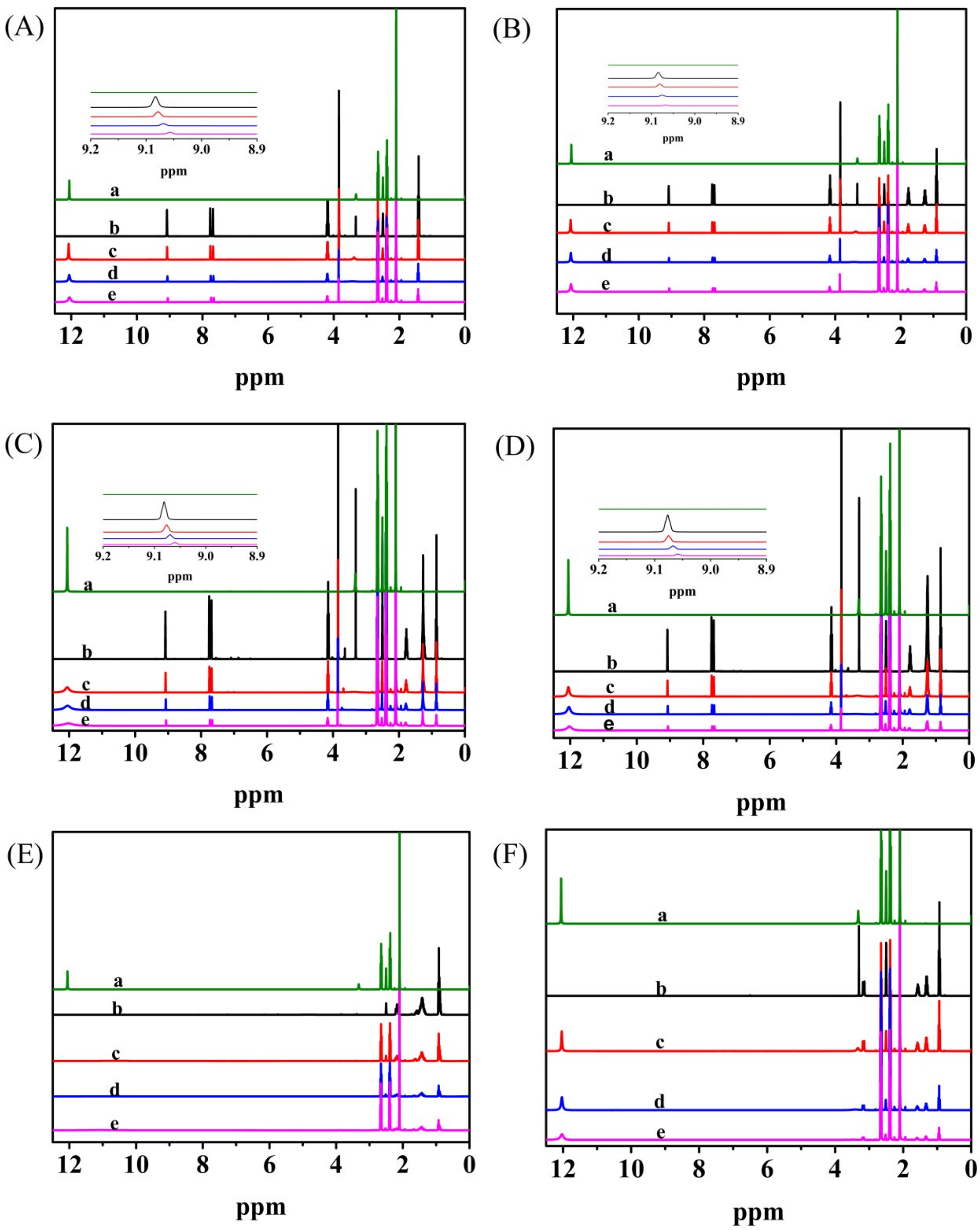

Fig. S14. ^{1}H NMR spectra of LA and different ILs. [Emim]BF_4 (A), [Bmim]BF_4 (B), [Hmim]BF_4 (C), [Omim]BF_4 (D), [TBP]BF_4 (E), and [TBA]BF_4 (F). (a and b represented LA and IL respectively. c, d, and e represented the mixture of IL and LA. The amount of IL was 12 mg and the amount of LA was 30 μL, 70 μL, and 110 μL in c, d and e, respectively. DMSO-d_6 was used as solvent.)

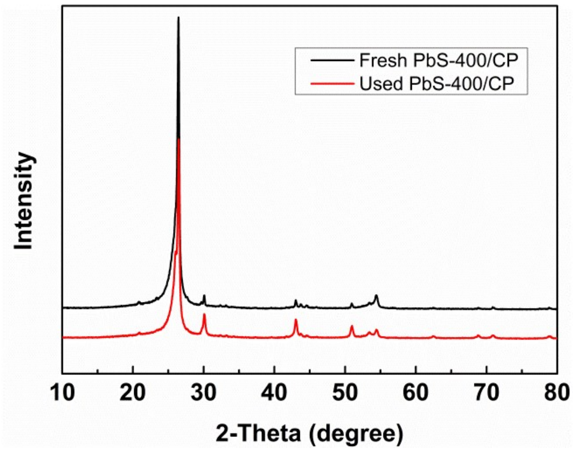

Fig. S15. XRD patterns of the fresh and used PbS-400/CP electrode

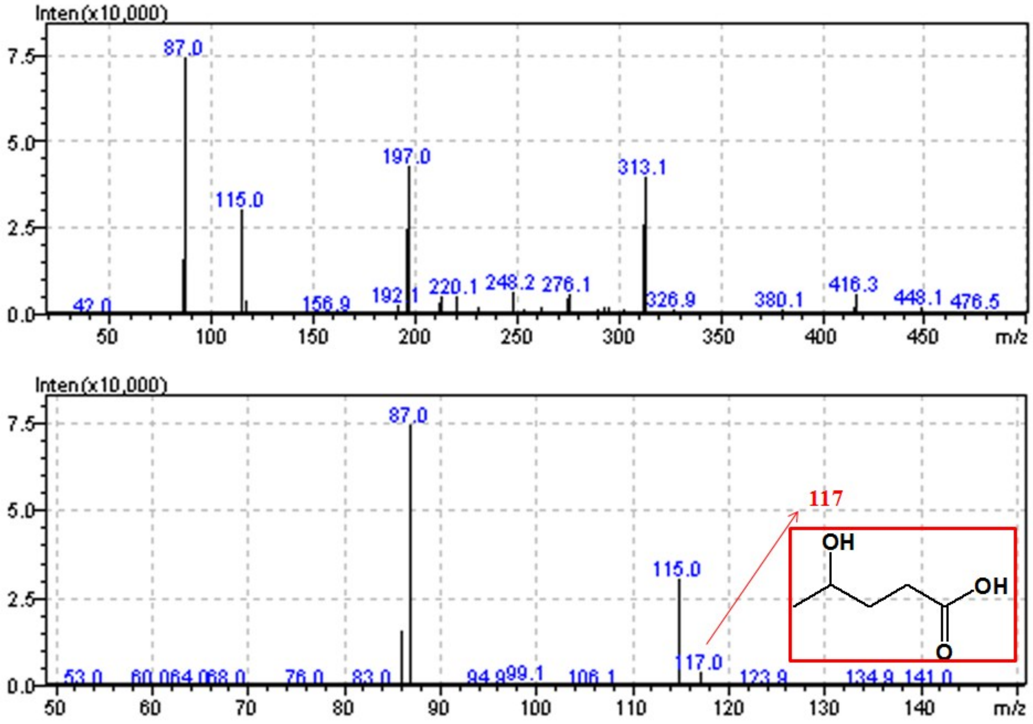

Fig. S16. MS spectra of the electrolyte after electrolysis for the electrochemical reduction of LA using PbS-400/CP in [Bmim]BF_4 (1.8 wt%)-MeCN-H_2O (1.8 wt%) at -2.15 V *vs.* Ag/Ag^+ for 4 h.

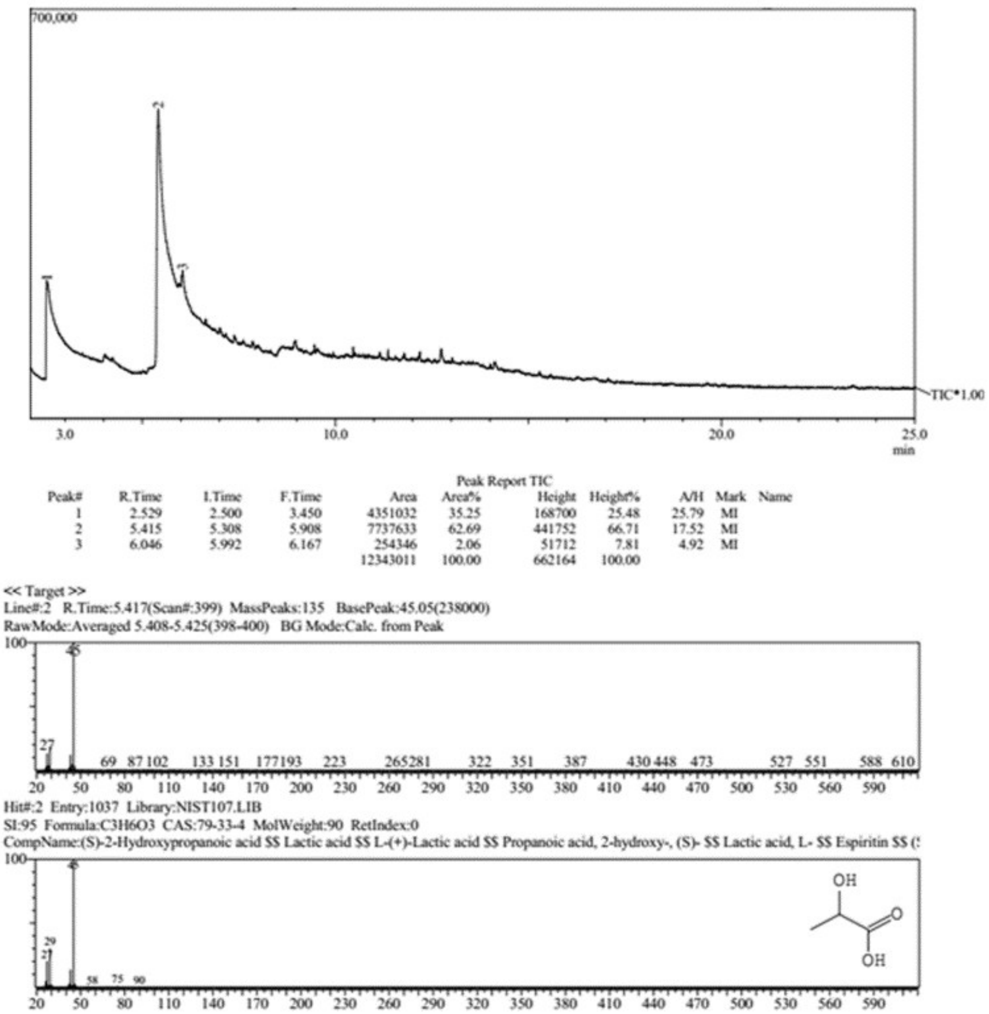

Fig. S17. GC-MS spectra of the electrolyte after electrolysis of pyruvic acid using PbS-400/CP in the electrolyte of [Bmim]BF_4 (1.8 wt%)-MeCN-H_2O (1.8 wt%) at -2.15 V *vs.* Ag/Ag^+ for 4 h.

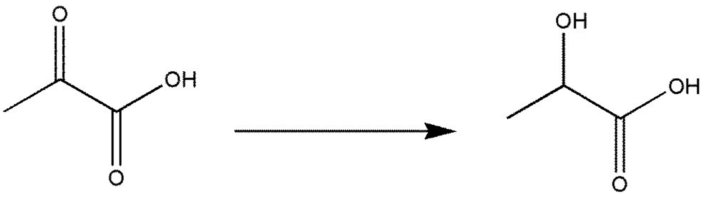

Scheme S1. Electrochemical reduction of pyruvic acid over PbS-400/CP electrode in the electrolyte of [Bmim]BF_4 (1.8 wt %)-MeCN-H_2O (1.8%).

Table S1. The chemical composition of the PbS-X.

Materials	The determined elements content from ICP-AES		Composition
	Pb (wt%)	S (wt%)	
PbS	86.54	12.69	PbS
PbS-300	85.81	13.04	$(PbS)_{28.06}/PbSO_4$
PbS-400	80.38	11.80	$(PbS)_{2.45}/PbSO_4$
PbS-500	70.42	10.26	$(PbS)_{0.16}/PbSO_4$
PbS-600	68.25	9.79	$PbSO_4$

The theoretical contents of Pb in PbS and $PbSO_4$ are 86.6 wt% and 68.3 wt%, respectively. The theoretical contents of S in PbS and $PbSO_4$ are 13.4 wt% and 10.57 wt%, respectively.
